# Resistin and Cardiac Arrest—A Prospective Study

**DOI:** 10.3390/jcm9010057

**Published:** 2019-12-25

**Authors:** Raluca M. Tat, Adela Golea, Rodica Rahaian, Ştefan C. Vesa, Daniela Ionescu

**Affiliations:** 1 Department of Anesthesia and Intensive Care I, “Iuliu Haţieganu” University of Medicine and Pharmacy, 400012 Cluj-Napoca, Romania; tatralu@yahoo.com; 2 Surgical Department of “Iuliu Haţieganu” University of Medicine and Pharmacy, 400012 Cluj-Napoca, Romania; 3 Department of Immunology Laboratory, County Emergency Hospital, 400006 Cluj-Napoca, Romania; 4 Department of Pharmacology, Toxicology and Clinical Pharmacology, “Iuliu Haţieganu” University of Medicine and Pharmacy, 400012 Cluj-Napoca, Romania; 5 Outcome Research Consortium, Cleveland, OH 44195, USA

**Keywords:** cardiac arrest, resistin, post-cardiac-arrest shock

## Abstract

The systemic response to ischemia-reperfusion that occurs after a cardiac arrest (CA) followed by the return of spontaneous circulation leads to endothelial toxicity and cytokine production, both responsible for the subsequent occurrence of severe cardiocirculatory dysfunction and early death. Resistin is emerging as a biomarker of proinflammatory status and myocardial ischemic injury and as a mediator of endothelial dysfunction. The study aimed to analyze the possible associations between several clinical and biological variables and the serum levels of resistin in CA survivors. Forty patients with out-of-hospital resuscitated CA, were enrolled in the study. Demographic, clinical and laboratory data (including serum resistin measurements at admission and at 6, 12, 24, 48 and 72 h) were recorded. For resistin, we calculated the area under the curve (AUC) using the trapezoidal method with measurements from 0 to 12 h, 0 to 24 h, 0 to 48 h and 0 to 72 h. Fifteen (37.5%) patients died in the first 72 h after CA. Cardiovascular comorbidities were present in 65% of patients. The majority of patients had post-CA shock (29 (72.5%)). Resistin serum levels rose in the first 12–24 h and decreased in the next 48–72 h. In univariate analysis, advanced age, longer duration of resuscitation, high sequential organ failure assessment score, high lactate levels, presence of cardiovascular comorbidities and the post-CA shock were associated with higher resistin levels. In multivariate analysis, post-CA shock or cardiovascular comorbidities were independently associated with higher AUCs for resistin for 0–12 h and 0–24 h. The only identified variable to independently predict higher AUCs for resistin for 0–48 h and 0–72 h was the presence of post-CA shock. Our data demonstrate strong independent correlation between high serum resistin levels, cardiac comorbidities and post-CA shock. The impact of the post-CA shock on serum concentration of resistin was greater than that of cardiac comorbidities.

## 1. Introduction

One of the relatively common presentations in the emergency department (ED) is that of a patient who suffered an out-of-hospital cardiac arrest (OHCA). Regardless of the etiology of cardiac arrest (CA), the physicians’ efforts are centered on the early control of all consequences secondary to the interruption of blood flow to organs and return of spontaneous circulation (ROSC). They are known under the term of post-cardiac-arrest syndrome (PCAS)—which is responsible for the high mortality of post-resuscitation patients [1,2]. In PCAS, four components have been described: post-cardiac-arrest brain injury, post-cardiac-arrest myocardial dysfunction, systemic response to ischemia/reperfusion and persistent precipitating pathology [2]. Although post-cardiac-arrest brain injury remains an important cause of mortality and morbidity among CA patients, the other elements of PCAS (like systemic response to ischemia/reperfusion) also lead to multiple organ failure and early death [2,3].

The pathophysiology of PCAS is very complex and involves ischemia-reperfusion injury and activation of nonspecific mechanisms of systemic inflammatory response. Summarizing the process, the oxygen supply during ischemia is reduced and the cellular metabolism is affected, ultimately resulting in an increase in the intracytoplasmic calcium concentration responsible for the first cellular and tissue lesions. During the reperfusion phase, following restoration of the blood flow, reactive oxygen species formed during the ischemic phase induce cell death through their cytotoxic effect (inactivation of cytochromes, alteration of membrane transport proteins, inducing lipid peroxidation of the membrane). The pro-oxidant state that occurs inside the cells marks the transition to the next stage, characterized by aggressive endothelial toxicity. The onset of vascular endothelial lesions paves the way to systemic inflammation via the ischemia-reperfusion mechanisms: cytokine production, complement activation, arachidonic acid synthesis, leukocyte adhesion to endothelial cells and triggering of activation and chemotaxis of polymorphonuclear neutrophils at the origin of the inflammatory response. All of these are responsible for the subsequent development of multiple organ failure. Of note, the activation of the systemic inflammatory response is also associated with changes in coagulation (intravascular coagulation dissemination), which generate additional endothelial lesions. This creates a vicious circle where inflammatory lesions and coagulation abnormalities induce further organ damage by accentuating pre-existing lesions and enhancing the persistence of the precipitating pathology of CA, more likely in close dependence with the duration of resuscitation and the rhythm of CA [4,5,6,7].

In the past few years, the research community has been focused on identifying biomarkers able to adequately predict the severity of the lesions that underline the pathophysiological processes in CA.

Resistin is a cysteine-rich, adipose-derived peptide hormone encoded by the *RETN* gene that is highly expressed in circulating monocytes, macrophages and vascular endothelium [8,9,10]. It is involved in numerous pathological processes (obesity, disorders of glucose and insulin metabolism, atherosclerosis, malignancies, rheumatic diseases, chronic kidney disease, etc.) [11,12,13,14]. Resistin has been suggested as a marker of the severity of myocardial ischemic lesion [8,12] and proposed as a mediator of endothelial dysfunction [8,12,15,16,17]. Moreover, resistin has been potentially introduced as a marker of proinflammatory status (cytokine-like) in relation to sepsis and in other nonseptic critical pathologies [8,12,13,14]. In a previous study, we investigated the role of resistin as a biomarker for predicting mortality after CA. The results showed that elevated serum resistin levels were highly predictive of mortality in critically ill patients who survived a CA [14,18].

Taking into account the proposed mechanisms of action of resistin and the pathophysiology of CA, the aim of our study was to investigate the clinical and biological variables that correlate with serum resistin levels in CA survivors.

## 2. Materials and Methods

A prospective, analytical, longitudinal, observational cohort study included consecutive patients resuscitated after the OHCA and admitted to the ED of County Emergency Hospital Cluj-Napoca between May 2016 and October 2017. Informed consent for inclusion in the study was obtained from patients’ proxies in all cases. The study was conducted in accordance with the Declaration of Helsinki, and the protocol was approved by the Ethics Committee of “Iuliu Haţieganu” University of Medicine and Pharmacy, with registration number 59/14.03.2016.

The inclusion criteria were as follows: age between 18–85 years and resuscitated OHCA. The exclusion criteria were as follows: ages under 18 or over 85 years, pregnancy, re-arrest with unsuccessful resuscitation within 6 h from hospital arrival, inmates, absence of informed consent and CA due to trauma, acute bleeding from nontraumatic condition, hypothermia or terminal neoplastic disease.

### 2.1. Study Protocol and Laboratory Assays

The management protocol of the patients admitted to the study, the post-CA shock definition and the lab protocols were previously described [18].

Each patient with out-of-hospital CA admitted to the study was resuscitated by emergency medical team members according to the recommendations of the European Resuscitation Council 2015 [19,20]. Fluids infusion and vasoactive drugs (adrenaline, noradrenaline, dopamine, dobutamine), alone or in combination, were administered in order to maintain mean arterial pressure ≥65 mmHg and urine output ≥0.5 mL/kg/h. For patients remaining comatose after successful resuscitation, able to maintain a systolic blood pressure above 90 mmHg (mean arterial pressure—MAP ≥65 mmHg) and without sepsis, controlled therapeutic hypothermia was administered in the first 24 h, in order to maintain a central temperature with a target between 34–35°C, using ice bags and cooling blanket. Reheating was slow, at a rate of 0.25–0.5 °C/h. According to local protocols which follow current guidelines, hyperthermia, seizures and hyperglycemia were avoided and immediately treated [21,22].

Blood samples were drawn from a peripheral vein where no medication was administered, at 0-time interval (emergency admission), 6, 12, 24, 48 and 72 h following resuscitation. Five-milliliter biochemistry vacutainers with serum separator clot activator were used for blood sample collection. To collect blood samples, we used 5 mL biochemistry vacutainers with serum separator clot activator. The identified hemolyzed samples were excluded and blood samples were immediately repeated. Samples were centrifuged at 3000 rotation/minutes during the first 60 min after collection and were stored at −70 °C. Subsequently, serum concentrations of biomarkers (resistin, S-100B and NSE) were analyzed using a quantitative sandwich immunoassay technique (ELISA; BioVendor, LM, Czech Republic) according to the manufacturer’s instructions. After processing, defrosted blood samples were no longer used and underwent destruction.

For every patient, the following data were recorded: demographic (age, gender), clinical (presence of cardiovascular diseases and/or strong risk factors for cardiovascular disease (arterial hypertension, coronary artery disease, valvular heart disease, congestive heart failure, history of stroke, diabetes mellitus and obesity), the rhythm of OHCA, duration of resuscitation, body mass index (BMI), presence of post-CA shock), sequential organ failure assessment (SOFA) score at admission and laboratory data (lactate and glycemia at admission; resistin at 6, 12, 24, 48 and 72 h). Obesity was defined by a body mass index (BMI) ≥30 kg/m^2^. The overweight was classified at a BMI between 25 and 29.9 kg/m^2^. Post-CA shock was defined as the need to administer vasoactive/inotropic therapy to maintain a MAP >65 mmHg for at least 6 h immediate after return of spontaneous circulation, although fluid therapy was adequate.

### 2.2. Statistical Analysis

Statistical analysis was performed using the MedCalc Statistical Software version 18.11.3 (MedCalc Software bvba, Ostend, Belgium; https://www.medcalc.org; 2019). Quantitative data normality was assessed using the Shapiro–Wilk test, measures of skewness and kurtosis and histograms. Quantitative data were expressed as median and interquartile range (IQR). Qualitative data were characterized by frequency and percentage. For resistin, we calculated the area under the curve (AUC) using the trapezoidal method with measurements from 0 to 12 h, 0 to 24 h, 0 to 48 h and 0 to 72 h. The sample size was calculated from a pilot study (13 patients with post-CA shock and 4 patients without post-CA shock). Calculated AUC for resistin, for 0–12 h measurements, showed a 24 ng × h/mL mean difference between the two groups. For a type 1 (a) error of 0.01 and a type 2 (b) error of 0.05, we calculated a sample size of 34 patients. The power of the study was calculated as 95%. Correlations between quantitative variables were assessed using the Spearman’s rank correlation coefficient. The differences between groups were verified with Mann–Whitney test. In order to find out which variables can be independently linked to resistin, we constructed several models using multiple linear regressions. Due to the fact that the resistin values followed a non-normal distribution, we performed a logarithmic transformation. We introduced the variables that were significantly associated with the AUCs for resistin during the univariate analysis. A *p*-value of less than 0.05 was considered statistically significant.

## 3. Results

Forty patients admitted to ED who met the inclusion criteria were included in the study. Patient characteristics are described in Table 1. On the first and second day, 12 (30%) patients died, and by the third day there were another three (7.5%) deaths. The 25 survivors after 72 h were followed for 30 days, and we recorded the deaths of 13 of them in this interval. Most of the recorded CA rhythm was asystole. Of the total number of patients admitted in our study, only 11 (28.5%) patients did not develop immediate post-resuscitation shock. Of the total admitted patients, 14 (35%) were obese and 20 (50%) were overweight.

For serum resistin levels we calculated the AUCs using the trapezoidal method with measurements from 0 to 24 h, 0 to 48 h and 0 to 72 h. Resistin levels and AUCs showed an increase in the first 12 h after admission, followed by a gradual decrease in the next 60 h (Table 2).

We found that SOFA score and serum lactate values at admission were the most important clinical and laboratory parameters associated with serum resistin levels (strong positive correlation to all repeated measurements) (Table 3). The serum resistin levels were not influenced by BMI.

The AUCs for resistin were higher in patients who presented asystole or PEA rhythm of CA (especially), cardiovascular comorbidities, history of congestive heart failure, arterial hypertension or post-CA shock (Table 4). We found no associations between AUCs for resistin and history of coronary artery disease, stroke, diabetes mellitus, obesity, smoking or alcoholic beverages.

Several models based on multiple linear regression were used in order to determine the independent association between clinical/laboratory data and the AUCs for resistin. The variables that were significantly linked to the AUCs in the univariate analysis were introduced in the models. Due to the fact that the resistin values followed a non-normal distribution, we performed a logarithmic transformation. When we introduced the history of congestive heart failure or arterial hypertension as separate variables, we found no statistically significant association with the log AUCs for resistin. Post-CA shock or cardiovascular comorbidities were independently associated with the log AUC for resistin for 0–12 h and 0–24 h. The only identified variable independently linked to the log AUC for resistin for 0–48 h and 0–72 h was the presence of post-CA shock (Table 5).

## 4. Discussions

CA involves the most severe form of circulatory failure. The complex changes produced by disruption of cell morpho-functional integrity during the general ischemia phase do not stop with the return of spontaneous circulation and are subsequently supplemented by those appearing during the reperfusion phase. The release of proinflammatory cytokines with the onset of systemic inflammatory response syndrome and endothelial damage (with coagulation/anticoagulation and fibrinogenesis/fibrinolysis imbalance) are intricate mechanisms that ultimately contribute to organ failures with negative impact prognosis of resuscitated patients [6,8,13,14,23].

In our previous study, we investigated for the first time the serum levels of resistin as a possible predictor of mortality after CA. Our results were promising, showing that high serum values of resistin accurately predicted death at 30 days, making resistin a marker with a high predictive value of survival [14,18].

However, resistin levels can be influenced by a variety of factors, such as the presence of atherosclerosis, obesity or sepsis. In light of this, we investigated the possible correlations of several clinical and biochemical factors with the serum concentration of resistin in patients with CA, for a better understanding of its role in CA [18].

Initially described in 1994 as a way of quantifying organ dysfunction by evaluating respiratory, cardiovascular, hepatic, renal, neurological and coagulation systems [24], the SOFA score remained useful over the years and is now being used with accuracy in quantifying the prognosis of critically ill patients [25]. The ischemia-reperfusion lesion is one of the most important mechanisms that link CA to multiple organ failures, including circulatory and cardiac dysfunction. Our results showed that there is a strong correlation between the severity of the disease (quantified by the SOFA score) and serum levels of resistin, a potential marker that may correctly reflect organ failures.

Over time, elevated levels of resistin have been associated with increased risk of coronary heart disease, especially with myocardial infarction (but not with stroke) [26] and with the degree of heart failure, both responsible for increasing the rate of cardiac events, including the risk of death [26,27]. At the same time, obesity, diabetes, high carbohydrate and unsaturated fat diet and chronic alcohol consumption, but not smoking, were described as cardiovascular risk factors correlated with elevated human serum resistin levels [28,29,30,31]. In our study, we found no associations between resistin levels in patients with CA and history of coronary artery disease, stroke, diabetes mellitus, obesity, smoking or alcoholic beverages. This reinforces the idea that in an acute critical illness high levels of resistin (or other adipokines) are mostly due to inflammatory status and not to adipose tissue mass or pre-existing unhealthy lifestyle [32].

At multivariate analysis, we found that the presence of post-CA shock and cardiovascular comorbidities were independently associated with serum resistin levels in the first 24 h after CA.

In fact, the presence of post-CA shock was the only independent variable associated with serum resistin levels at 48 and 72 h following CA. These results show that the elevated serum concentrations of resistin might be influenced by the post-CA shock, rather than by pre-CA cardiovascular comorbidities. The shock that occurs after CA is the result of myocardial dysfunction, vasoplegic shock and systemic inflammatory response [25]. Part of a complex vicious circle, as this shock becomes more refractory to treatment, cardiocirculatory dysfunction evolves in turn into a more severe form, resulting in multiple organ failures responsible for early death. The strong association of resistin with post-CA shock and with the presence of cardiovascular comorbidities may support the theory that serum resistin levels correlate equally with both the amplitude of the inflammatory process and cardiac dysfunction after resuscitation.

In previous studies, increased serum levels of lactate upon admission to the emergency department and intensive care units were associated with the negative prognosis of patients with acute critical illness [18]. Our data revealed that high serum levels of lactate at admission correlate strongly with serum resistin levels. This may support the idea that resistin is directly involved in the process of systemic inflammation in CA pathogenesis, seeing as elevated lactate levels are in fact associated with severe cardiocirculatory dysfunction [33]. However, at multivariate analysis, the aforementioned correlation did not remain statistically significant, suggesting that there were other important factors that interfere in the CA physiopathological sequence.

To our knowledge, this is the first study that evaluated the factors that influence the kinetics of resistin after CA. These results were obtained on a small number of patients, although statistically significant. The high number of measurements present an accurate kinetics of resistin after a CA, with a peak at 12–24 h and a rapid decrease to admission values after 48 h. This is important for future studies on acute events, as it shows that the focus on resistin should be especially in the first 24 h.

In order to strengthen our hypothesis, it is essential that we develop further/future studies on larger groups of patients. Also, they must include other markers of acute inflammation, with a special interest for those reportedly correlated with resistin during acute cardiovascular events: tumor necrosis factor α (TNF-α), interleukin-6 (IL-6), high-sensitivity C-reactive protein (hs-CRP) and other proinflammatory cytokines [34]. Resistin promotes the production of TNF-α, IL-1β, IL-6 and other cytokines [12,35]. There are several drugs that have been shown to reduce the levels of resistin in chronic administration: statins, anti-TNF-α monoclonal antibodies and folic acid [36,37,38]. Experimental animal or in vitro studies in acute situations with drugs that lower resistin concentration are worth considering.

Other markers that could provide insights into the functions and pathophysiological implications of resistin are the microvesicles (large extracellular vesicles that appear from different cells after apoptosis) [39]. Platelet-derived microvesicles are a source of TNF-α and IL-6, while endothelial-derived microvesicles are stimulated by TNF-α [40]. Elevated levels of endothelial-derived microvesicles were found in acute coronary syndrome patients, but they were not evaluated in patients that survived a CA; one can speculate that investigating this class of micro-vesicles will offer valuable data [41,42].

Markers that evaluate post-cardiac-arrest myocardial dysfunction should be studied in any future research on patients after a successfully resuscitated CA. Left ventricular systolic dysfunction is present in almost 60% of patients resuscitated after CA [33]. The assessment of left ventricle ejection fraction, biomarkers of ventricular dysfunction and the correlation with proinflammatory markers will generate a better understanding of the complexity of PCAS. The N-terminal pro-B-type natriuretic peptide (NT-proBNP) and marinobufagenin are reliable indicators of ventricular dysfunction and, as such, can serve as excellent candidates for future studies on OHCA [43,44].

Even though the multivariate analysis showed that the post-CA shock was independently associated with higher levels of resistin, an important bias could be the presence of cardiovascular comorbidities. The myocardial systolic and diastolic dysfunctions appear in post-CA shock, even if the patient does not have a prior coronary disease [45]. Future studies should include patients with noncardiac causes of CA, because the presence of cardiac diseases aggravates the left ventricular dysfunction. Other diseases that were proven to have an influence on the resistin concentrations should be excluded (nonalcoholic fatty liver disease, asthma, autoimmune disease, chronic kidney disease) [46]. That could provide a clearer picture about the association between post-CA shock and resistin kinetics.

## 5. Conclusions

Our findings demonstrate strong independent correlation between high serum resistin levels, cardiac comorbidities and post-CA shock. The impact of the post-CA shock on serum concentration of resistin was greater than that of cardiac comorbidities.

## Figures and Tables

**Table 1:** Baseline characteristics of the study group.

Characteristics		Eligible Patients with CA (*n* = 40)
**Age, years, median (IQR)**		67 (59.2 to 76.0)
**Gender, *n* (%)**	**Female**	12 (30.0)
	**Male**	28 (70.0)
**Presenting rhythm, *n* (%)**	**Asystole**	23 (57.5)
	**PEA**	5 (12.5)
	**VF**	11 (27.5)
	**VT without pulse**	1 (2.5)
**Duration of CPR, minutes, median (IQR)**		15 (7.7 to 28.7)
**Current smoking, *n* (%)**		4 (10)
**Chronic alcohol consumer, *n* (%)**		5 (12.5)
**Medical history, *n* (%)**	**Non-cardiovascular comorbidities**	18 (45.0)
	**Cardiovascular comorbidities**	26 (65.0)
	**Arterial hypertension**	23 (57.5)
	**Coronary artery disease**	17 (42.5)
	**Valvular heart disease**	8 (20%)
	**Congestive heart failure**	15 (37.5)
	**Stroke**	3 (7.5)
	**Diabetes mellitus**	7 (17.5)
**BMI, median (IQR)**		28 (26.0 to 31.0)
**Obesity, *n* (%)**		14 (35)
**SOFA score, median (IQR)**		15 (12.0 to 16.0)
**Patients with post-CA shock, *n* (%)**		29 (72.5)
**Lactate (mmol/L), median (IQR)**		10.42 (7.6 to 12.9)
**Blood glucose (mg/dL), median (IQR)**		249.0 (156.0 to 330.0)

IQR = interquartile range; PEA = pulseless electrical activity; VF = ventricular fibrillation; VT = ventricular tachycardia; CPR = cardiopulmonary resuscitation; BMI = body mass index; SOFA = sequential organ failure assessment score; CA = cardiac arrest.

**Table 2:** Median serum levels of resistin and the AUC for resistin during the first 72 h.

Variable		Median (IQR)
**Resistin, (ng/mL)**	**at 0 h**	7.1 (4.6 to 11.8)
	**at 6 h**	9.8 (4.4 to 17.7)
	**at 12 h**	13.5 (5.5 to 21.0)
	**at 24 h**	12.3 (6.7 to 21.0)
	**at 48 h**	7.2 (3.5 to 14.6)
	**at 72 h**	7.4 (3.6 to 11.9)
**AUC resistin, (ng × h/mL)**	**in the first 12 h**	26.0 (11.5 to 43.2)
	**in the first 24 h**	25.8 (15.2 to 44.7)
	**in the first 48 h**	16.6 (10.4 to 35.1)
	**in the first 72 h**	34.6 (17.9 to 46.5)

AUC = area under the curve; IQR = interquartile range.

**Table 3:** Correlations between the AUCs for resistin and the study quantitative variables.

Variable	AUC for 0–12 h		AUC for 0–24 h		AUC for 0–48 h		AUC for 0–72 h	
	r	*p*	r	*p*	r	*p*	r	*p*
**Age, years**	0.316	0.04	0.360	0.03	0.467	0.01	0.356	0.08
**Duration of CPR, minutes**	0.364	0.02	0.386	0.02	0.414	0.02	0.357	0.08
**BMI**	0.039	0.8	–0.148	0.4	–0.183	0.3	–0.141	0.5
**SOFA score**	0.586	<0.001	0.579	<0.001	0.510	0.006	0.529	0.007
**Lactate (mmol/L)**	0.499	<0.001	0.592	<0.001	0.501	0.007	0.509	0.009
**Blood glucose (mg/dL)**	0.185	0.2	0.417	0.01	0.176	0.3	–0.023	0.9

AUC = area under the curve; CPR = cardiopulmonary resuscitation; BMI = body mass index; SOFA = sequential organ failure assessment score; r = correlation coefficient.

**Table 4:** Associations between the AUC for resistin and the qualitative variables studied.

Variable		AUC for 0–12 h		AUC for 0–24 h		AUC for 0–48 h		AUC for 0–72 h	
		Median (IQR)	*p*	Median (IQR)	*p*	Median (IQR)	*p*	Median (IQR)	*p*
**Gender**	**Female**	26.5 (23.0 to 42.5)	0.4	30.0 (17.4 to 44.1)	0.8	20.6 (13.3 to 49.2)	0.3	25.0 (17.1 to 74.0)	0.7
	**Male**	23.0 (10.2 to 43.2)		25.2 (13.8 to 44.7)		15.9 (9.3 to 33.8)		35.3 (18.7 to 46.1)	
**Presenting rhythm of CA**	**Asystole/** **PEA**	30.5 (22.0 to 47.7)	0.002	30.3 (19.9 to 51.4)	0.002	23.5 (14.5 to 38.7)	0.009	37.8 (25.0 to 69.8)	0.01
	**VF/VT without pulse**	10.5 (4.2 to 22.5)		14.3 (8.0 to 24.1)		12.4 (4.8 to 16.0)		23.4 (13.8 to 31.9)	
**Cardiovascular comorbidities**	**present**	29.0 (22.0 to 45.5)	0.03	37.2 (18.3 to 50.2)	0.01	22.7 (14.2 to 38.1)	0.06	37.4 (23.6 to 64.0)	0.08
	**absent**	16.5 (4.7 to 31.5)		18.8 (11.1 to 25.8)		13.7 (5.3 to 28.4)		27.6 (15.0 to 36.7)	
**History of arterial hypertension**	**present**	28 (22 to 45)	0.1	37.2 (18.2 to 49.1)	0.05	22 (14.1 to 39)	0.1	37.8 (22.2 to 67.3)	0.1
	**absent**	18 (7 to 37)		19.9 (14.3 to 26.4)		14.5 (5.8 to 27.3)		29.3 (15.4 to 36.9)	
**History of congestive heart failure**	**present**	30.5 (23 to 46.5)	0.04	38 (29.9 to 49.7)	0.02	34.8 (19.6 to 46.4)	0.02	52.8 (23.5 to 92)	0.04
	**absent**	18.5 (7.5 to 32.7)		19.3 (9.8 to 31.6)		14.5 (9.2 to 23.9)		27.6 (16.9 to 36.8)	
**Post-CA shock**	**present**	31.0 (24.0 to 47.5)	<0.001	30.3 (24.1 to 51.4)	<0.001	30.8 (15.9 to 38.7)	0.002	41.8 (23.4 to 70.8)	0.01
	**absent**	10.0 (4.0 to 15.0)		13.4 (8.0 to 18.8)		12.4 (4.8 to 15.8)		27.5 (12.1 to 34.6)	

AUC = area under the curve; IQR = interquartile range; PEA = pulseless electrical activity; VF = ventricular fibrillation; VT = ventricular tachycardia; CA = cardiac arrest.

**Table 5:** Multiple linear regression for the AUCs for resistin.

**Variables for the log of AUC for 0–12 h**	**B**	***p***	**95.0% CI for B**	
			**Min**	**Max**
(Constant)	0.784	<0.001	0.581	0.988
Post-CA shock	0.528	<0.001	0.309	0.747
Cardiovascular comorbidities	0.214	0.04	0.009	0.419
**Variables for the log of AUC for 0–24 h**	**B**	***p***	**95.0% CI for B**	
			**Min**	**Max**
(Constant)	0.954	<0.001	0.768	1.140
Post-CA shock	0.415	<0.001	0.211	0.619
Cardiovascular comorbidities	0.201	0.04	0.004	0.397
**Variables for the log of AUC for 0–48 h**	**B**	***p***	**95.0% CI for B**	
			**Min**	**Max**
(Constant)	0.939	<0.001	0.739	1.139
Post-CA shock	0.470	0.001	0.212	0.727
**Variables for the log of AUC for 0–72 h**	**B**	***p***	**95.0% CI for B**	
			**Min**	**Max**
(Constant)	1.321	<0.001	1.154	1.488
Post-CA shock	0.303	0.01	0.079	0.526

AUC = area under the curve; B = standardized beta coefficient; CA = cardiac arrest.
